# Hepatitis B virus in the Lao People’s Democratic Republic: a cross sectional serosurvey in different cohorts

**DOI:** 10.1186/1471-2334-14-457

**Published:** 2014-08-23

**Authors:** Antony P Black, Phonethipsavanh Nouanthong, Naphavan Nanthavong, Chanthasone Souvannaso, Keooudomphone Vilivong, Prapan Jutavijittum, Bounthome Samountry, Nina Lütteke, Judith M Hübschen, Sylvie Goossens, Fabrice Quet, Yves Buisson, Claude P Muller

**Affiliations:** Lao-Lux Laboratory, Institut Pasteur du Laos, Vientiane, Laos; Institute of Immunology, Centre de Recherche Public de la Santé/Laboratoire National de Santé, 20A, rue Auguste Lumière, L-1950 Luxembourg, Grand-Duchy Luxembourg; Institut de la Francophonie pour la Médecine Tropicale, Vientiane, Laos; Department of Pathology, Faculty of Medicine, Chiang Mai University, Chiang Mai, Thailand; Department of Pathology, Faculty of Medicine, University of Health Sciences, Vientiane, Laos; Independent consultant, Vientiane, Laos

**Keywords:** Hepatitis B virus, Serology, Vaccine, Cross-sectional

## Abstract

**Background:**

Despite hepatitis B vaccination at birth and at 6, 10 and 14 weeks of age, hepatitis B virus (HBV) infection continues to be endemic in the Lao People’s Democratic Republic (PDR). We carried out a cross-sectional serological study in infants, pre-school children, school pupils and pregnant women to determine their burden of disease, risk of infection and vaccination status.

**Methods:**

A total of 2471 participants between 9 months and 46 years old were recruited from urban (Vientiane Capital, Luang Prabang), semi-urban (Boulhikhamxai and Savannakhet) and remote rural areas (Huaphan). All sera were tested for anti-HBs and anti-HBc. Sera testing positive for anti-HBc alone were further tested for the presence of HBsAg.

**Results:**

A low prevalence of HBsAg (0.5%) was detected among infants from Vientiane and Luang Prabang, indicating some success of the vaccination policy. However, only 65.6% had protective anti-HBs antibodies, suggesting that vaccination coverage or responses remain sub-optimal, even in these urban populations.

In pre-school children from remote areas in Huaphan, 21.2% were positive for anti-HBc antibodies, and 4.6% were for HBsAg positive, showing that a significant proportion of children in these rural regions have early exposure to HBV. In pre-school children with 3 documented HBV vaccinations, only 17.0% (15/55) were serologically protected.

Among school-children from semi-urban regions of Luang Prabang, Boulhikhamxai and Savannakhet provinces, those below the age of 9 who were born after HBV vaccine introduction had anti-HBc and HBsAg prevalence of 11.7% and 4.1%, respectively. The prevalence increased to 19.4% and 7.8% of 10–14 year olds and to 27% and 10.2% of 15–19 year olds.

Pregnant women from Luang Prabang and Vientiane had very high anti-HBc and HBsAg prevalence (49.5% and 8.2%), indicating high exposure and risk of onward vertical transmission to the unborn infant.

**Conclusions:**

Overall, the results demonstrate a dramatic deficiency in vaccination coverage and vaccine responses and/or documentation within the regions of Lao PDR studied, which included urbanized areas with better health care access. Timely and effective hepatitis B vaccination coverage is needed in Lao PDR.

**Electronic supplementary material:**

The online version of this article (doi:10.1186/1471-2334-14-457) contains supplementary material, which is available to authorized users.

## Background

Hepatitis B virus (HBV) infects more than one third of the world’s population and there are an estimated 360 million chronic carriers worldwide, with a high risk for cirrhosis, hepatocellular carcinomas and liver failure [1, 2]. Half of the global HBV-attributable deaths occur in the WHO Western Pacific Region which includes the Lao People’s Democratic Republic (PDR) [3]. The prevalence of HBV infection is extremely heterogeneous between and within countries in the WPR. The WHO regional committee has recommended a target to reduce the prevalence of chronic hepatitis B infections among children aged younger than 5 years to less than 1% by 2017 [4–6].

HBV is endemic in the Lao PDR, about 8% of blood donors being chronic carriers of HBV, and is the cause of high morbidity and mortality [7, 8]. It is believed that most infections occur during early childhood, e.g. during birth or early family life [9]. If infected at birth, children have a 90% risk of developing chronic infection. This rate decreases to 30% if infected between the ages of one and five and to 5-10% if infected after the age of 5 [10]. As screening for hepatitis B surface antigen (HBsAg) in pregnant women and immunoglobulin prophylaxis in newborns are not widely practiced in Lao PDR, routine infant vaccination is the mainstay of the prevention of early childhood infection.

From 2001, there has been a phased introduction of HBV vaccine into the national immunization schedule. Currently, infants are scheduled to receive the HBV birth dose within 24 hours after birth, followed by HBV containing vaccine at 6, 10 and 14 weeks of age in combination with diphtheria, tetanus, pertussis and *Haemophilus influenzae* b vaccine (DTP- HepB-Hib) [11–13]. The birth dose of HBV vaccine, followed by the timely 3 dose schedule, is assumed 70 to 95% effective in preventing mother-to-child transmission of HBV [4, 14–16]. However, the HBV birth dose coverage in Lao PDR was only approximately 28% in 2011–2012 [13], largely because the majority of births are unattended home-births (62% in 2010 [17]). A recent study showed that even among infants born at health-care facilities, the birth dose vaccination coverage was only 74%. The study also showed that missing stock and misconceptions around vaccine contraindications were major issues that should be addressed. In addition, the authors highlighted a need to strengthen HepB birth dose inclusion in home birthing and post-natal services and increase the percentage of medically attended home-births and postnatal home visits [16]. Furthermore, due to the difficult access to healthcare for a large proportion of the Lao population, only 78% of children less than 1 year old received all three DTP-HepB-Hib vaccinations in 2011 [17].

Currently there are no serological studies in young people and pregnant women in Lao PDR. To better understand the epidemiology of hepatitis B and the impact of immunisation on the disease, we performed a cross-sectional survey in four different sub-populations from Lao PDR with different ages and vastly different access to vaccination and general health care; infants, pre-school children, school pupils and pregnant women.

## Methods

### Study population

We recruited participants from 5 different provinces in Lao PDR; Luang Prabang in the north of the country, Huaphan in the northeast, Vientiane and Boulhikhamxai in the centre and Savannakhet in the south (Table 1). The study was approved by the Laos National Ethics Committee (reference NECHR 001/2011for infants, school students and pregnant women; reference 2013–732 for pre-school children in Huaphan province). As none of the participant selection was systematically randomised, we cannot exclude different selection bias in the different groups.

**Table 1:** **Participant profile**

Participants	Infants (9 months; Luang Prabang and Vientiane Capital)	Pre-school aged children (1–4 years; Huaphan)	School children (5–19 years; Luang Prabang, Boulhikhamxai, Savannakhet)	Pregnant women (16–46 years; Luang Prabang and Vientiane Capital)	Total
**Male**	93	72	835	0	1015
**Female**	104	60	909	398	1492
**Total**	197	132	1744	398	2471
**N° tested**	192	132	1689	388	2401

### Infants

Infants aged 9–16 months were recruited following maternal informed consent. All 197 enrolled infants were attending vaccination clinics in Luang Prabang Provincial hospital or the Vientiane Mother and Child Central Hospital on predetermined days in 2011.

### Pre-school aged children

A total of 132 pre-school aged children (1–4 years) were recruited from rural settings in Huaphan province Xamtai and Kuan districts, in 2013. Mothers were informed by the village chief of the date and place of the upcoming study. All mothers that attended the recruitment site with their children and gave informed consent were enrolled. Out of 415 parents informed about the study, only 132 gave consent for their child’s enrollment due to the very complicated living conditions in this rural setting.

Immunization status was determined from certificates of vaccination (yellow cards) when available. Nutritional status was estimated by weight/height ratio and medium upper left arm circumference (MUAC). Villages were defined as being remote from health centres when the travel time exceeded 100 minutes by the most rapid means of transport.

### School pupils

School pupils (n = 1744) aged 5–19 years participating in a 2011 national immunization campaign against measles and rubella were recruited from semi-urban areas of the following provinces; Luang Prabang, Boulhikhamxai (Paksan and Pakading districts) and Savannakhet (Kaisone and Outhoumphone districts).

### Pregnant women

All pregnant women that attend the antenatal units in Luang Prabang Provincial hospital or the Vientiane Mother and Child Central Hospital on predetermined days in 2011 were asked to participate. A total of 398 women aged 16–46 years provided informed written consent and were enrolled in the study.

### Serological testing

After obtaining informed consent from the parents or participants, venous blood was drawn. Serum was isolated by centrifugation and stored at -80°C until testing. Commercially available enzyme immunoassay kits (Diasorin, Italy) were used for the detection of HBsAg (Murex HBsAg kit) and antibodies against hepatitis B surface antigen (anti-HBs; EIT-AB-AUK3 kit) and core antigen (anti-HBc; Murex anti-HBc kit). HBsAg was tested only in samples that were anti-HBc positive and anti-HBs negative. Although this strategy may miss some subjects with early acute or low level HBV infection and immune tolerance, these individuals are rare and previous studies estimate that we will detect more than 99% of HBsAg positive individuals [7]. Positivity for HBsAg was considered indicative of chronic infection although a small percentage may be acute phase infections. Anti-HBs indicate a post-infectious or post-vaccination immunity according to the presence or absence of anti-HBc. In the latter case, the anti-HBs alone were considered protective if greater than 10 mIU/mL. Anti-HBc alone were interpreted as a likely marker of previous exposure to the virus. Individuals negative for all HBV serological markers were considered seronegative. Data were analysed by Chi square or Fischer exact test, as appropriate, using GraphPad Prism and STATA software. All the data were analysed anonymously.

## Results

**Figure 1:**
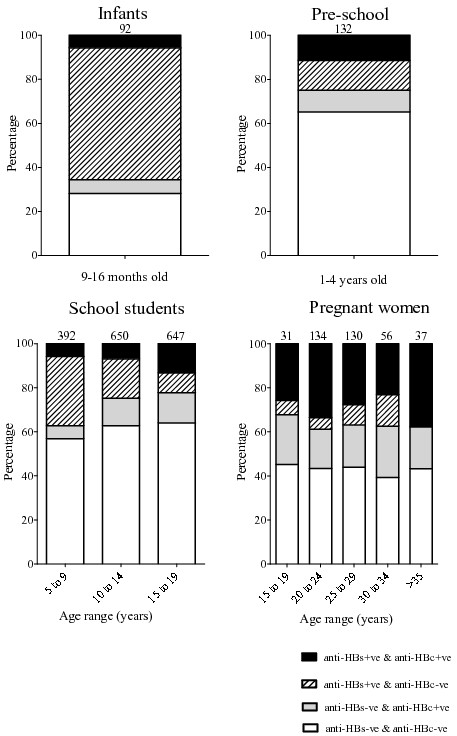
**Seroprevalence of anti-HBs and anti-HBc antibodies according to age.** Numbers above bars represent the number of participants per age group. These data exclude those who had equivocal results.

Seventy sera gave uninterpretable /equivocal results for anti-HBs antibodies and were excluded from further analysis. Of the remaining 2401 samples, 294 (12.2%) were positive for anti-HBc alone, 459 (19.1%) for anti-HBs alone and 296 (12.3%) for both anti-HBc and anti-HBs while 1352 (56.3%) were negative for all serum markers. HBsAg positive samples represented 7.2% of all individuals and 58.5% of anti-HBs negative, anti-HBc positive participants (Figure 1 and Table 2).

**Table 2:** **HBV serology in infants, school pupils and pregnant women according to location**

	Infants N (%)			Pre-school children N (%)	School pupils N (%)				Pregnant women N (%)		
	VTN	LPB	Total	Huaphan	LPB	BLX	SVN	Total	VTN	LPB	Total
**anti-HBs- anti-HBc-**	23 (24.5)	31 (31.6)	54 (28.1)	86 (65.1)	318 (53.4)	358 (63.9)	369 (69.2)	1045 (61.9)	98 (51.9)	69 (34.7)	167 (43.0)
**anti-HBs + anti-HBc -**	61 (64.9)	54 (55.1)	115 (59.9)	18 (13.6)	131 (22.0)	108 (19.3)	58 (10.9)	297 (17.6)	6 (3.2)	23 (11.6)	29 (7.5)
**anti-HBs– anti-HBc +**	7 (7.5)	5 (5.1)	12 (6.3)	13 (9.9)	61 (10.2)	57 (10.2)	75 (14.1)	193 (11.4)	30 (15.9)	46 (23.1)	76 (19.6)
**anti-HBs + anti-HBc+**	3 (3.2)	8 (8.2)	11 (5.7)	15 (11.4)	86 (14.4)	37 (6.6)	31 (5.8)	154 (9.1)	55 (29.1)	61 (30.7)	116 (29.9)
HBsAg + *	1 (1.1)	0	1 (0.5)	6 (4.5)	50 (8.4)	20 (3.6)	63 (11.8)	133 (7.9)	18 (9.5)	14 (7.0)	32 (8.2)
**Total**	94	98	192	132	596	560	533	1689	189	199	388

*only tested on anti-HBs negative - anti-HBc positive samples.

*Abbreviations used*: VTN Vientiane, LPB Luang Prabang, BLX Bolikhamxai and SVN Savannaket.

### Infants

In total, 132 infant samples from Luang Prabang and Vientiane were tested. Overall anti-HBc prevalence was 12.0% with 6.3% positive for anti-HBc alone. Anti-HBs antibodies alone were detected in 59.9%. Both anti-HBc and anti-HBs were detected in 5.7% of infants. Anti-HBs negative, unprotected infants represented 34.4% of all tested. Only 0.5% of infants were HBsAg positive (Figure 1 and Table 2).

### Pre-school aged children

Anti-HBc and anti-HBs antibody prevalence in pre-school aged children from remote areas of Huaphan province was 21.2% and 25.0%, respectively. Thus, 75% of the pre-school children remained anti-HBs negative and susceptible to infection. Anti-HBc antibodies alone were detected in 9.9% of pre-school aged children and anti-HBs alone in 13.6% of children with 11.4% positive for both antibodies. HBsAg was detected in 4.6% of the children (Figure 1 and Table 2). Only 66 (50%) were able to present their certificate of vaccination (yellow card). Protective anti-HBs antibodies were detected in 19.7% of participants without vaccination records and 30.3% of those with vaccination records; 15 of the 55 children (27.2%) with records showing 3 doses of DTP-HepB-Hib vaccine and in one of the 14 children (7.1%) who received a birth dose of HepB vaccine followed by 3 doses of DTP-HepB-Hib (Table 3). Prevalence of anti-HBs alone, indicative of vaccination, was 19.5% in villages close to health centres as compared to 4.0% in remote villages (p = 0.01). Stunted and underweight children comprised 51.5% and 21.2% of those with protective levels of anti-HBs antibodies respectively as compared to 53.5% and 27.3% of those without anti-HBs (not significant). The prevalence of acute malnutrition, as measured by weight/ height and MUAC was 3% and 6% in those with protective anti-HBs titres compared to 3% and 10.1% in those without anti-HBs (not significant). HBsAg was detected in 4.5% of the pre-school children.

**Table 3:** **Vaccination and serological status of pre-school children from Huaphan province**

Vaccine received as documented on yellow card		Children who received vaccine N (%)	Children with anti-HBs antibodies N (%)
**No HepB birth dose**	DTP-HepB-Hib 1	9	5 (55.6%)
	DTP-HepB-Hib 2	2	0 (0%)
	DTP-HepB-Hib 3	41	14 (34.5%)
**Received HepB birth dose**	DTP-HepB-Hib 1	0	0
	DTP-HepB-Hib 2	0	0
	DTP-HepB-Hib 3	14	1 (7.1%)

### School children

In total, 20.5% of the school children were anti-HBc positive and 26.7% were anti-HBs positive. Only 9.1% were double positive for both markers; 11.4% and 17.6% were single positive for anti-HBc and anti-HBs, respectively. In school children aged 9 years and under, anti-HBc and anti-HBs were 11.7% and 37.2%, respectively. HBsAg prevalence in this age-group was 4.1%. School children aged between 10 and 14 years had anti-HBc, anti-HBs and HBsAg prevalence of 19.4%, 24.8%, and 7.8%, respectively. Those between the ages of 15 and 19 years had anti-HBc, anti-HBs and HBsAg prevalence of 27.0%, 22.3%, and 10.2%, respectively (Figure 1 and Table 2).

HBsAg prevalence was significantly higher among those from Savannaket (11.8%) than from Boulhikhamxai (3.6%) or Luang Prabang (8.4%; p < 0.05). The average age of school pupils did not significantly differ between Savannakhet (14.6 years), Luang Prabang (11.7 years) and Boulhikhamxai (12.6 years).

### Pregnant women

Anti-HBc prevalence was 49.5% in pregnant women; 19.6% were anti-HBc positive alone and 29.9% were double positive for anti-HBc and anti-HBs. Anti-HBs prevalence was 37.4%; 7.5% for anti-HBs alone without anti-HBc. HBsAg was detected in 8.2% of the pregnant women in this study (Figure 1 and Table 2).

## Discussion

Among the 192 infants aged 9 to16 months tested in the urban settings of Vientiane and Luang Prabang, only one carried HBsAg while 23 (12%; all were aged 9–10 months) were anti-HBc positive. Anti-HBc positivity, usually indicative of HBV exposure, may also be attributed to passive transfer of maternal antibodies still detectable at this age. For instance, in African children aged 3 months to 6 years, the seroprevalence of anti-HBc was 44.4% during the first 6 months of age and still 18.8% at 12 months [7, 18]. Importantly, the low HBsAg prevalence in the infants in our study may be seen as a positive impact of the vaccination policy implemented in the districts. This is also supported by the 59.9% seroprevalence rate of anti-HBs alone, reflecting an active, albeit low-level, immunization campaign in these areas. The three doses of HBV vaccine against hepatitis B were introduced in the EPI of the Lao PDR in 2001 and the birth dose was added in 2003 [13, 17]. Consequently, all infants included in our study should have received 4 doses of vaccine against HBV and be anti-HBs positive. However, our results show that vaccination coverage among infants even in the main cities of Luang Prabang and Vientiane remains inadequate. This is in line with the low vaccination coverage with the HBV birth dose (44-48%) and the DTP-HepB-Hib3 (80-85%) reported from these cities in 2011 [13, 17]. It can be expected that in a rural setting the coverage is even lower.

Indeed only 13.6% of our pre-school children aged 1 to 4 years from rural settings in Huaphan province had anti-HBs antibodies alone, suggesting that very few children had been vaccinated. Distance from the nearest health centre was significantly associated with vaccination status, emphasizing the relationship between access and vaccination coverage (p = 0.01). In contrast 21.3% were anti-HBc positive, reflecting a prior exposure to HBV in this age group rather than passive transfer of maternal antibodies. Furthermore, 4.5% were HBsAg carriers as a result of early life exposure. That only 27% of 55 children who received three doses of DTP-Hib-HepB vaccine according to their yellow vaccination cards had protective levels of anti-HBs antibodies is of further concern. Altered immunogenicity of the vaccine because of deficiencies in the cold chain [9, 19] or because of expired batches may be one explanation. In our study a high percentage of the children were stunted (low height for ages) and underweight (low weight for age). However, we did not find a lower vaccine response in malnourished children as compared to those who were not malnourished, similar to the findings of previous studies [20]. Unreliable certification of vaccination in the yellow cards by the mobile teams would be another explanation of major concern.

The school pupils in our study were recruited from semi-urban settings in Luang Prabang, Boulhikhamxai and Savannakhet provinces. Those aged 9 and under were born after the introduction of the HBV vaccines. The HBsAg prevalence increased from 4.3% (similar to the 4.5% in the under-fives in Huaphan province) in the 5 to 9 years old to 7.8% between 10 and 14 years. Assuming that most chronic infections are acquired during the first years of life, the lower HBsAg prevalence can be considered as the result of an improved vaccination coverage. The increase of anti-HBc prevalence from 19.4% in the 10 to 14 years old to 27.0% in the 15 to 19 years bracket shows that HBV exposure continues and may even intensify after the age of 14 years. The higher HBsAg prevalence in the province of Savannakhet is of note. This cannot be explained by age difference and is perhaps a reflection of variable vaccination coverage between provinces. As we had no vaccination histories for the school pupils, we could not address this issue further. The high HBsAg prevalence that we detect is in contrast to a recent nationwide study which reported low prevalence (1.7%) in children aged 5–9 years of age [21]. We mainly attribute these differences to a possible lower sensitivity of the rapid test used in the latter study or logistical differences such as sampling methodology.

The pregnant women in our study were recruited from urban areas of Luang Prabang and Vientiane. The high HBsAg prevalence of 8.2% in this cohort represents a high risk of vertical transmission of HBV and stresses the need to promote HBV vaccination of all children at birth, a strategy that has proved effective in many other countries within the region [22, 23]. This prevalence is in accordance with published studies [7, 8] and unpublished data from the Lao Red Cross that show between 8–10% of adult Lao blood donors nationwide are HBsAg positive. However, it is in contrast to the 2.9% HBsAg positivity in mothers aged 15–49 years in the aforementioned nationwide survey [21]. Regional HBsAg prevalence varies between and within countries, according to vaccination coverage and other factors. Thus, studies in Vietnam, Cambodia and southern China have also reported high adult HBsAg prevalences of 7 to 20% [22–26].

## Conclusions

Strengthening the policy for preventing mother to child transmission of HBV in Lao PDR is needed to reach the objective of reducing the prevalence of HBsAg carriage below 1% in children aged less than 5 years. We gave examples from rural and semi-urban settings in 5 different provinces that sub-optimal coverage of the HBV vaccine birth dose since its introduction in 2003 threatens the possibility of achieving this objective. Our study suggests some major shortcomings of the outreach programme including low vaccination coverage but also problems with vaccine responses and/or flawed vaccination cards. Another obstacle to this important public health intervention is that the majority of children are born at home largely without medical attention indicating that the “dose at birth” is probably given too late or not at all in particular in remote rural areas. We also highlight that even in areas best-supported by the health system, exposure to HBV among unvaccinated individuals occurs during secondary school and continues throughout adulthood.

Lao PDR is heterogeneous in terms of vaccination coverage and access to healthcare. For example infants attending health facilities in urban areas have better access to vaccination than children from rural settings who comprise the largest proportion in Lao PDR. Because of geographical and ethnic heterogenicity and non-randomised sampling it is difficult and even not indicated to extrapolate our seroprevalence data to the entire Lao population. Nevertheless, we clearly show that there is a positive impact of HBV vaccine as evidenced by low prevalence of HBsAg in infants and school-children under 10 years i.e. those born after its introduction.

Therefore strengthening hygienic interventions or providing an additional HBV vaccine dose at 10 years of age should be considered. A significant improvement in immunization coverage of children would also require strengthening routine outreach services, by mobile teams, especially in remote areas, paired with a strict management of the cold chain and reliable certification of vaccinations.

## Authors’ original submitted files for images

Below are the links to the authors’ original submitted files for images. Authors’ original file for figure 1

## Abbreviations

HBc: Hepatitis B core antigen
HBV: Hepatitis B virus
HBsAg: Hepatitis B surface antigen
MUAC: Medium upper arm circumference
DTP-HepB-Hib: Diphtheria- tetanus-pertussis-hepatitis B-haemophilus influenzae B containing vaccine.

## References

[1] Lavanchy D (2004). Hepatitis B virus epidemiology, disease burden, treatment, and current and emerging prevention and control measures. J Viral Hepat.

[2] WHO (2000). Hepatitis B fact sheet.

[3] Clements CJ, Baoping Y, Crouch A, Hipgrave D, Mansoor O, Nelson CB, Treleaven S, van Konkelenberg R, Wiersma S (2006). Progress in the control of hepatitis B infection in the Western Pacific Region. Vaccine.

[4] WHO (2007). Western Pacific Regional Plan for Hepatitis B control through immunization.

[5] WHO (2011). Progress towards meeting the 2012 hepatitis B control milestone: WHO Western Pacific Region, 2011. Wkly Epidemiol Rec.

[6] WHO (2013). Hepatitis B control through vaccination: setting the target. Regional committee.

[7] Jutavijittum P, Andernach IE, Yousukh A, Samountry B, Samountry K, Thammavong T, Keokhamphue J, Toriyama K, Muller CP (2013). Occult hepatitis B infections among blood donors in Lao PDR. Vox Sang.

[8] Jutavijittum P, Yousukh A, Samountry B, Samountry K, Ounavong A, Thammavong T, Keokhamphue J, Toriyama K (2007). Seroprevalence of hepatitis B and C virus infections among Lao blood donors. Southeast Asian J Trop Med Public Health.

[9] Pan CQ, Duan ZP, Bhamidimarri KR, Zou HB, Liang XF, Li J, Tong MJ (2012). An algorithm for risk assessment and intervention of mother to child transmission of hepatitis B virus. Clin Gastroenterol Hepatol.

[10] Goldstein ST, Zhou F, Hadler SC, Bell BP, Mast EE, Margolis HS (2005). A mathematical model to estimate global hepatitis B disease burden and vaccination impact. Int J Epidemiol.

[11] WHO (2012). National immunisation data - EPI summaries by country.

[12] Program LI, Health LM (2007). Comprehensive multi-year plan: 2007–2011. National Immunisation Program. Lao People’s Democratic Republic.

[13] (LSB) MoHMaLSB (2012). Lao Social Indicator Survey (LSIS) 2011–12.

[14] Lee C, Gong Y, Brok J, Boxall EH, Gluud C (2006). Effect of hepatitis B immunisation in newborn infants of mothers positive for hepatitis B surface antigen: systematic review and meta-analysis. BMJ.

[15] Wong VC, Ip HM, Reesink HW, Lelie PN, Reerink-Brongers EE, Yeung CY, Ma HK (1984). Prevention of the HBsAg carrier state in newborn infants of mothers who are chronic carriers of HBsAg and HBeAg by administration of hepatitis-B vaccine and hepatitis-B immunoglobulin. Double-blind randomised placebo-controlled study. Lancet.

[16] Centers for Disease C, Prevention (2013). Hepatitis B vaccine birthdose practices in a country where hepatitis B is endemic - Laos, December 2011-February 2012. MMWR Morb Mortal Wkly Rep.

[17] Department of Planning and International Cooperation M, Lao PDR (2011). National Health Statistic Report FY 2010–2011: contributing to monitor Millenium Development Goals.

[18] Rey-Cuille MA, Njouom R, Bekondi C, Seck A, Gody C, Bata P, Garin B, Maylin S, Chartier L, Simon F, Vray M (2013). Hepatitis B virus exposure during childhood in Cameroon, central african republic and senegal after the integration of HBV vaccine in the expanded program on immunization. Pediatr Infect Dis J.

[19] Dent E, Selvey CE, Bell A, Davis J, McDonald MI (2010). Incomplete protection against hepatitis B among remote Aboriginal adolescents despite full vaccination in infancy. CDI Quarterly Report.

[20] Rey-Cuille MA, Seck A, Njouom R, Chartier L, Sow HD, Mamadou, Ka AS, Njankouo M, Rousset D, Giles-Vernick T, Unal G, Sire JM, Garin B, Simon F, Vray M (2012). Low immune response to hepatitis B vaccine among children in Dakar, Senegal. PLoS One.

[21] Xeuatvongsa A, Komada K, Kitamura T, Vongphrachanh P, Pathammavong C, Phounphenghak K, Sisouk T, Phonekeo D, Sengkeopaseuth B, Som-Oulay V, Ishii K, Wakita T, Sugiyama M, Hachiya M (2014). Chronic hepatitis B prevalence among children and mothers: results from a nationwide, population-based survey in Lao People’s Democratic Republic. PLoS One.

[22] Ol HS, Bjoerkvoll B, Sothy S, Van Heng Y, Hoel H, Husebekk A, Gutteberg T, Larsen S, Husum H (2009). Prevalence of hepatitis B and hepatitis C virus infections in potential blood donors in rural Cambodia. Southeast Asian J Trop Med Public Health.

[23] Nguyen TH, Vu MH, Nguyen VC, Nguyen LH, Toda K, Nguyen TN, Dao S, Wannemuehler KA, Hennessey KA (2013). A reduction in chronic hepatitis B virus infection prevalence among children in Vietnam demonstrates the importance of vaccination. Vaccine.

[24] Nguyen VT, McLaws ML, Dore GJ (2007). Highly endemic hepatitis B infection in rural Vietnam. J Gastroenterol Hepatol.

[25] Chen P, Yu C, Ruan B, Yang S, Ren J, Xu W, Luo Z, Li L (2013). Prevalence of hepatitis B in insular regions of southeast China: a community-based study. PLoS One.

[26] Hipgrave DB, Nguyen TV, Vu MH, Hoang TL, Do TD, Tran NT, Jolley D, Maynard JE, Biggs BA (2003). Hepatitis B infection in rural Vietnam and the implications for a national program of infant immunization. Am J Trop Med Hyg.

[CR27] The pre-publication history for this paper can be accessed here:http://www.biomedcentral.com/1471-2334/14/457/prepub

