# Supplementary figures and images for: Hepatitis B virus in the Lao People’s Democratic Republic: a cross sectional serosurvey in different cohorts
Source: BMC Infect Dis. 2014 Aug 23;14:457. doi: 10.1186/1471-2334-14-457 (PMC4158128; doi:10.1186/1471-2334-14-457)

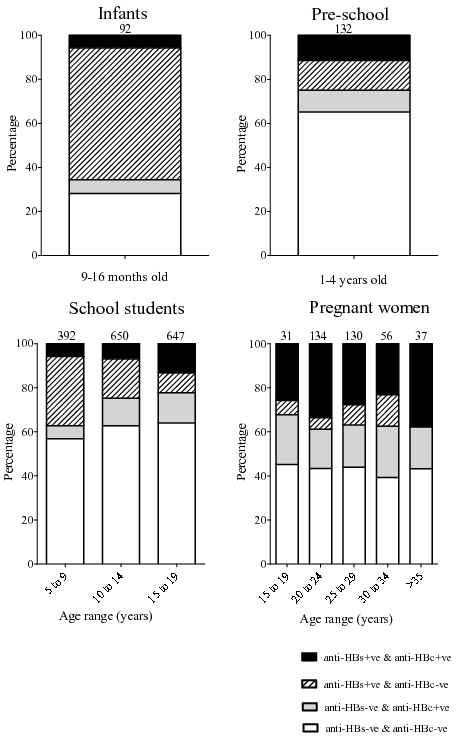

Supplement: Supplementary file 1 — Authors’ original file for figure 1 [file 12879_2014_3762_MOESM1_ESM.png]
